# Single-cell-based system to monitor carrier driven cellular auxin homeostasis

**DOI:** 10.1186/1471-2229-13-20

**Published:** 2013-02-04

**Authors:** Elke Barbez, Martina Laňková, Markéta Pařezová, Alexis Maizel, Eva Zažímalová, Jan Petrášek, Jiří Friml, Jürgen Kleine-Vehn

**Affiliations:** 1 Department of Plant Systems Biology, VIB and Department of Plant Biotechnology and Genetics, Ghent University, 9052, Gent, Belgium; 2 Department of Applied Genetics and Cell Biology, University of Natural Resources and Life Sciences, Vienna (BOKU), 1190, Vienna, Austria; 3 Institute of Experimental Botany, The Academy of Sciences of the Czech Republic, 16502, Praha 6, Czech Republic; 4 Department of Stem Cell Biology, Center for Organismal Studies, University of Heidelberg, 69120, Heidelberg, Germany; 5 Department of Functional Genomics and Proteomics, Faculty of Science, and CEITEC, Masaryk University, Kamenice 5, CZ-62500, Brno, Czech Republic

**Keywords:** Auxin homeostasis, DR5, Auxin carrier, Auxin transport

## Abstract

**Background:**

Abundance and distribution of the plant hormone auxin play important roles in plant development. Besides other metabolic processes, various auxin carriers control the cellular level of active auxin and, hence, are major regulators of cellular auxin homeostasis. Despite the developmental importance of auxin transporters, a simple medium-to-high throughput approach to assess carrier activities is still missing. Here we show that carrier driven depletion of cellular auxin correlates with reduced nuclear auxin signaling in tobacco Bright Yellow-2 (BY-2) cell cultures.

**Results:**

We developed an easy to use transient single-cell-based system to detect carrier activity. We use the relative changes in signaling output of the auxin responsive promoter element DR5 to indirectly visualize auxin carrier activity. The feasibility of the transient approach was demonstrated by pharmacological and genetic interference with auxin signaling and transport. As a proof of concept, we provide visual evidence that the prominent auxin transport proteins PIN-FORMED (PIN)2 and PIN5 regulate cellular auxin homeostasis at the plasma membrane and endoplasmic reticulum (ER), respectively. Our data suggest that PIN2 and PIN5 have different sensitivities to the auxin transport inhibitor 1-naphthylphthalamic acid (NPA). Also the putative PIN-LIKES (PILS) auxin carrier activity at the ER is insensitive to NPA in our system, indicating that NPA blocks intercellular, but not intracellular auxin transport.

**Conclusions:**

This single-cell-based system is a useful tool by which the activity of putative auxin carriers, such as PINs, PILS and WALLS ARE THIN1 (WAT1), can be indirectly visualized in a medium-to-high throughput manner. Moreover, our single cell system might be useful to investigate also other hormonal signaling pathways, such as cytokinin.

## Background

The phytohormone auxin is crucial to control plant growth and development. At the cellular level, auxin regulates cell division, cell expansion, and cellular differentiation [1]. Auxin largely exerts its action through a multistep signaling pathway: Aux/IAA proteins are repressors of the AUXIN RESPONSE FACTOR (ARF) transcription factors. Auxin directly binds to the nuclear co-receptors TRANSPORT INHITOR RESPONSE/AUXIN F-BOX PROTEIN (TIR/AFB) and the Aux/IAA. Auxin binding causes the subsequent degradation of Aux/IAA transcriptional repressors [2-4]. Subsequently, auxin perception leads to the de-repression of the ARF transcription factors, initiating transcriptional reprogramming.

The spatial and temporal distribution of auxins depends on auxin metabolism (biosynthesis, conjugation, and degradation) and the activity of cellular auxin transporters [5]. To date, various auxin carriers have been identified [6], among which the most prominent are auxin influx carriers of the AUXIN RESISTANT1/LIKE AUX1 (AUX/LAX) class, ABC transporters of the MULTIDRUG RESISTANCE (B-type) subfamily, and PIN-FORMED (PIN) auxin carriers [7-9]. Pharmacological and genetic interference with auxin carriers have illustrated the importance of auxin transport mechanisms for various aspects of plant development [10]. In particular, classical auxin transport inhibitors, such as 1-naphthylphthalamic acid (NPA) [11-13], are valuable tools to assess various auxin carrier-mediated developmental processes. Typically, auxin carriers mediate the cellular auxin import or export at the plasma membrane and, thus, regulate the auxin availability for nuclear auxin signaling (carrier-driven cellular auxin homeostasis). However, recently, a subclass of PIN proteins, such as PIN5 and PIN8, has been shown to reside at the endoplasmic reticulum (ER) and to control cellular auxin homeostasis presumably via the regulation of intracellular auxin compartmentalization into the ER lumen [14-16]. Yet another evolutionary distinct PIN-LIKES (PILS) putative auxin carrier family functions at the ER, indicating broad developmental and evolutionary importance of intracellular auxin transport [17-19].

The transport capacity of a multitude of auxin carriers and their sensitivity to auxin transport inhibitors has been analyzed in plant cell systems, such as *Arabidopsis* protoplasts or Bright Yellow-2 (BY-2) cell cultures of tobacco (*Nicotiana tabacum*), and in heterologous cell systems, such as yeast and mammalian cells [9,14,20-23]. These elaborate auxin transport assays are important tools to study transport activities and mechanisms. However, it would be desirable to develop easier methods to asses auxin carrier activity. An alternative approach has been proposed for the indirect visualization of the carrier-driven auxin homeostasis [24,25]. This bioassay utilizes the stimulating effect of free auxin levels on root hair elongation. The root hair-specific expression of an auxin carrier and its action on the root hair length is used to indirectly visualize carrier driven auxin homeostasis. However, auxin fluxes in neighboring tissues also contribute to the regulation of root hair growth [26], preventing the combined use of ectopic carrier expression and its sensitivity to auxin transport inhibitors. Moreover, the time-consuming generation of stable transgenic lines might limit the use of this bioassay for high-throughput applications.

Another frequently used tool to monitor auxin signaling is the synthetic, highly auxin responsive promoter DR5, created by tandem repeats of the auxin responsive element (AuxRE) from the soybean GH3 promoter [27]. Previously, the DR5 promoter activity has been suggested to indicate the relative rate of nuclear auxin signaling in various tissues [28-33]. DR5 has been used to visualize auxin signaling maxima and minima which, however, do not correlate in all cells with the actual auxin levels possibly due to cell type-dependent cues [32,34,35].

Here, we present a novel single cell based system, using the DR5 promoter, to address cellular mechanisms that affect cellular auxin homeostasis and ultimately auxin signaling. We show the correlation between DR5 promoter activity and fluctuations in cellular auxin levels in tobacco BY-2 cells. Our data suggest that the transient expression of *DR5rev*:*monomeric RED FLUORESCENT PROTEIN* (*mRFP*) reporter for nuclear auxin signaling can be used to illustrate the relative state of cellular auxin signaling in tobacco BY-2 cells. By means of this single-cell-based system, the relative auxin carrier induced changes in cellular auxin signaling were monitored, indirectly indicating auxin carrier activity. As a proof of concept, we assessed the prominent PIN auxin carrier activity and confirmed their effects on cellular auxin homeostasis/signaling [14,21]. Moreover, a pharmacological approach revealed that the activity regulation of PIN2 at the plasma membrane and PIN5 at the ER are distinct. Furthermore, we show that this single-cell-based system could be analogously used to investigate other putative carriers, such as PILS and WAT1, or potentially even other hormonal pathways, such as cytokinin.

## Results

### Indirect visualization of auxin carrier activity in tobacco BY-2 cells

In previous studies, the synthetic auxin-responsive promoter element DR5 fused to the monomeric *RFP* or *GFP* reporter gene (*DR5rev*:*mRFP*/*GFP*) [30,36,37] was used to visualize the auxin response maxima within tissues and it was proposed to indirectly estimate auxin distribution [29,30,33]. However, auxin distribution and DR5-based auxin signaling does not always correlate in plant tissues presumably due to cell type-dependent cues [32].

**Figure 1:**
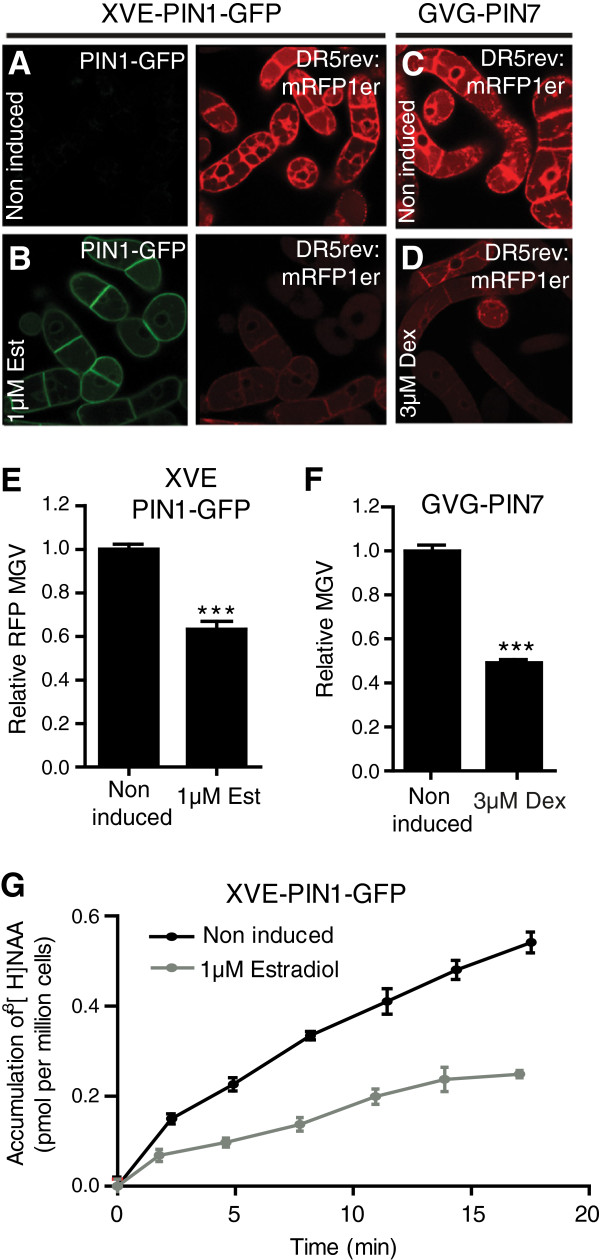
**Correlation between altered DR5 promoter activity and cellular auxin accumulation in stably transformed tobacco BY-2 cells.** Tobacco BY-2 cells stably transformed with the auxin responsive *DR5rev*:*mRFP* and the estradiol (Est) inducible *XVE*-*PIN1*-*GFP* or the dexamethasone (Dex) inducible *GVG*-*PIN7* construct. (**A**,**B**) Induction of *PIN1*-*GFP* (in green) with estradiol visibly decreases DR5rev:mRFP (in red) signal intensity compared to non-induced cells as visualized by confocal imaging. (**C**,**D**) Dexamethasone-dependent induction of *PIN7* visibly decreases DR5rev:mRFP signal intensity compared to non-induced cells. (**E**) Graph depicts mean gray values (MGV) of the DR5rev:mRFP signal intensity in *PIN1* induced and non-induced cells (n = 150). (**F**) MGV of the DR5rev:mRFP signal intensity in *PIN7* induced and non-induced cells (n = 150). (**G**) [^3^H]NAA accumulation assays in non-induced and estradiol-induced (*XVE*-*PIN1*-*GFP*) cells. Error bars represent standard error. Statistical significance was evaluated with the unpaired student T-test (* P < 0.05, ** P < 0.01, *** P < 0.0001).

To reduce cell type dependent effects, we tested whether the DR5 promoter could be used in tobacco BY-2 cell cultures to indirectly estimate auxin carrier activity. In order to address the correlation between auxin carrier activity and DR5 promoter activity in tobacco BY-2 cells, we stably transformed the *DR5rev*:*mRFP* construct into transgenic BY-2 lines carrying the construct for inducible *PIN1GFP*[38] or *PIN7*[21] expression. PIN1 and PIN7 are plasma membrane localized auxin efflux carriers important for plant growth and development [6,21]. Induction of *PIN1GFP* and *PIN7*, which causes cellular auxin depletion [21], decreased DR5rev:mRFP signal intensity compared to non-induced cells, reflecting lower nuclear auxin signaling (Figure 1A–1F, Additional file 1: Figure S1A). The DR5rev:RFP signal intensity is represented by the average mean gray value (MGV) of the induced cell population (n > 150 single cells) relative to the average MGV of the uninduced control population (n > 150).

Within the estradiol induced *PIN1*-*GFP* expressing BY-2 cell population, we observed a negative correlation between DR5rev:mRFP and PIN1-GFP signal intensity (Additional file 1: Figure S1B and S1C), suggesting lower levels of nuclear auxin signaling in case of high PIN1-GFP activity.

To unambiguously depict auxin carrier activity of PIN1-GFP and PIN7, we performed auxin accumulation assays on the same cell lines and observed lower accumulation of the radiolabelled synthetic auxin 1-naphtylacetic acid ([^3^H]NAA) in estradiol induced *PIN1*-*GFP* expressing cells compared to non-induced cells (Figure 1G). Our findings indicate that DR5rev:mRFP signal intensity (Figure 1A–F) correlates with cellular auxin accumulation (Figure 1G), presumably due to carrier induced changes in cellular auxin content and subsequent alterations in auxin signaling.

We conclude that under our experimental conditions the DR5 promoter activity can be used in BY-2 cells to indirectly visualize auxin carrier-dependent regulation of cellular auxin homeostasis.

### Procedure for transient auxin carrier expression in a single-cell-based system

In BY-2 cells, the visualization of auxin signaling could be used to indirectly monitor carrier driven cellular auxin homeostasis. To establish a medium-to-high throughput assay, we elaborated on procedures to transiently express auxin carriers. Particle bombardment is an easy to use procedure that enables high transformation efficiencies at low plasmid concentrations and has, in case of partial automatization, the potential for high-throughput use [39,40]. We adjusted the previously described particle bombardment procedure [39,40] for efficient, transient tobacco BY-2 transformation (see Materials and methods). To obtain high protein expression levels, BY-2 cell cultures in the exponential growth phase were used. DNA concentrations ranging from 0.05 μg/μl to 1 μg/μl were sufficient for transient expression and resulted in a correlation between DNA concentration and expression levels, allowing fine-tuning of the gene expression (data not shown). The co-transformation efficiency was tested by transformation of two plasmids at different concentration ratios and the co-transformation levels were calculated (Additional file 1: Table S1). Although dependent on concentration, in general the co-transformation efficiency was very high (approximately 90% at 0.05 μg/μl for both plasmids), enabling the two plasmids to be efficiently co-transformed (Additional file 1: Table S1). Thus, BY-2 particle bombardment can be used as a suitable method to efficiently co-express genes of interest.

### Transient single-cell-based-system to monitor auxin signaling

**Figure 2:**
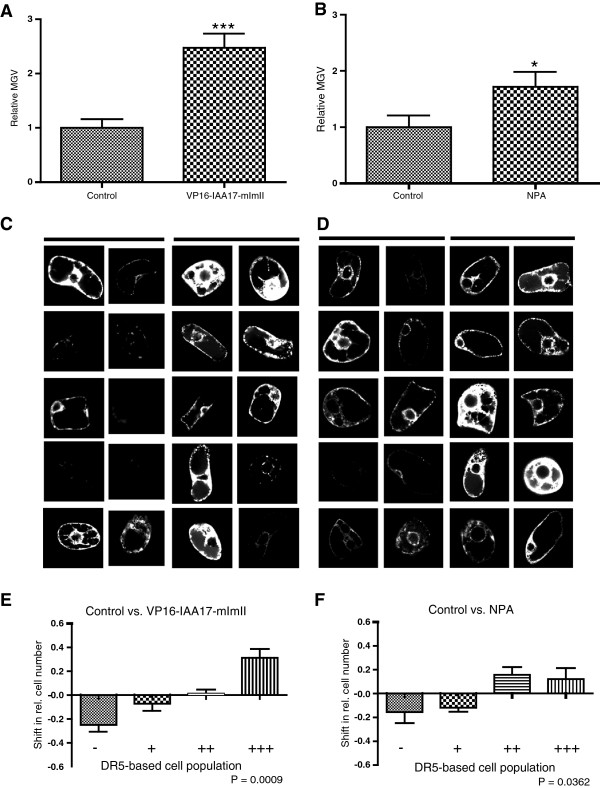
**Effect of altered auxin signaling and transport capacity on cellular auxin homeostasis.** (**A**,**B**) Graphs represent the relative average mean gray values (MGV) of the DR5rev:mRFP signal intensity. Error bars represent standard error (n = 60). Statistical significance was evaluated with the unpaired student T-test (* P < 0.05, ** P < 0.01, *** P < 0.0001). (**A**) Coexpression of *DR5rev*:*mRFP* and the stabilized version of *IAA17* fused to a VP16 activator domain (*35S*:*VP16*-*IAA17mImII*), causing constitutive auxin signaling, significantly increased the relative average MGV compared to the *35S*:*HDEL*-*GFP*-expressing control cells. (**B**) NPA treatment leads to an increased MGV/DR5 signaling compared to transformants maintained in standard cultivation medium. (**C**) 10 representative pictures are shown for the control cells (left panel) and the cells overexpressing *VP16*-*IAA17*-*mImII* (right panel). (**D**) 10 representative pictures are shown for the untreated control cells (left panel) and the cells treated with NPA (right panel). (**E**,**F**) Graphs depict the changes in relative number of transformed cells displaying a low (-), medium (+), high (++), and very high (+++) DR5rev:mRFP signal between the two samples addressing *VP16*-*IAA17*-*mImII* expression or NPA treatment (for detailed description of the quantification, see Additional file 1: Figure S2). Error bars represent standard error (n = 3 repetitions with at least 50 counted cells). Statistical significance was evaluated with the ANOVA test; the P-value is indicated.

To initially test whether the transient DR5 expression in BY-2 cells could be used to visualize qualitative differences in levels of auxin signaling between two samples of interest, we transiently co-transformed tobacco BY-2 cells with the auxin responsive *DR5rev*:*mRFP* construct and the stabilized auxin signaling repressor *IAA17mImII* fused to the activator domain of the herpes simplex virus VP16. This construct leads to constitutive auxin signaling in plant cells [41,42]. As expected, the mean gray value (MGV) of DR5rev:mRFP (reflecting auxin signaling) was higher in cells co-transformed with *DR5rev*:*mRFP* and *35S*:*VP16IAA17mImII* than in control cells expressing *DR5rev*:*mRFP* and the inert endoplasmic reticulum (ER) marker *35S*:*HDELGFP* (Figure 2A–2C). To further elaborate on the relative changes in DR5/auxin signaling, we subdivided the transformed cell population in 4 classes according to the relative MGV. Individual cells were scored as low (-) with a relative MGV below 0.5 (2^-1^), medium (+) with a relative MGV above 0.5 (2^-1^) and below 1 (2^0^), high (++) with a relative MGV between 1 (2^0^) and 2 (2^1^) and very high (+++) with a relative MGV value higher than 2 (2^1^). (Figure 2E and Additional file 1: Figure S2). This alternative visualization allows us to trace the shifts in relative cell numbers with low, medium, high and very high RFP signal intensity between two samples and to compare even more subtle differences in DR5rev:mRFP signal intensities. 35S:VP16-IAA17mImII enhanced auxin signaling in our system and accordingly reduced the relative cell numbers with low/medium and increased the cell numbers with strong, or very strong RFP signal intensity (Figure 2E).

These findings indicate that the transient *DR5rev*:*mRFP* expression in BY-2 cells can be used to monitor the qualitative differences in nuclear auxin signaling.

Next, we examined whether our single-cell-based system can be used to address mechanisms of auxin transport. Therefore we treated the *DR5rev*:*mRFP*-transformed cell population with the auxin transport inhibitor NPA that reduces cellular auxin efflux and, hence, increases cellular auxin levels [43]. As expected, NPA treatment significantly increased the relative rate of DR5 signaling in our transient assay (Figure 2B, 2D and 2F), revealing that NPA action on cellular auxin efflux and cellular auxin homeostasis can be monitored in our single-cell-based system.

Our findings suggest that the DR5- and single-cell-based system can be used to qualitatively monitor changes in auxin signaling. However, in order to use this system in a meaningful way, the experimental design needs to be carefully chosen, because DR5 activity has been suggested not to solely reflect auxin signaling. The phytohormone brassinolide affects the expression of several auxin responsive genes as well as the activity of the DR5 promoter in *Arabidopsis thaliana*[34,35] (Additional file 1: Figure S3A and S3B). Nevertheless, in our experimental conditions, brassinolide treatment did not increase the average MGV of *DR5rev*:*mRFP* transformed tobacco BY-2 cells (Additional file 1: Figure S3C–S3E). This finding indicates that either brassinosteroids do not increase auxin signaling in BY-2 cells or that our approach is not sensitive enough to trace subtle differences, such as brassinosteroid-induced auxin signaling.

### Auxin carrier trafficking and localization in the single-cell-based system

To further assess the usability of the method, we studied the effect of the PIN auxin carrier activity on the cellular auxin signaling. Initially, a time-frame of presumably high PIN protein activity was defined by investigating the PIN trafficking/localization after transient BY-2 transformation. Transmembrane proteins, such as PIN proteins, are co-translationally inserted into the ER membrane. Plasma membrane-localized PIN proteins, such as PIN1, are exported subsequently from the ER and sorted to the plasma membrane [44]. In contrast, PIN5 proteins remain at the ER membrane, where they function as regulators of (intra)cellular auxin homeostasis [14].

**Figure 3:**
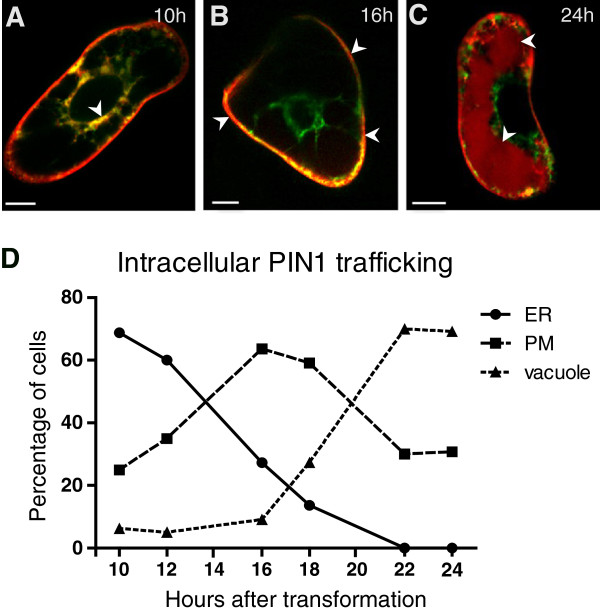
**Cellular localization and trafficking of PIN1**-**RFP.** (**A**) Colocalization of PIN1 proteins (in red) with the ER marker HDEL-GFP (in green) 10 h after transformation. (**B**) PIN1-RFP preferentially localizes to the plasma membrane (PM) after 16 h. (**C**) 24 h after transformation, PIN1-RFP shows vacuolar localization indicating lytic turnover. (**D**) Relative number of cells showing pronounced PIN1-RFP signal in the ER, PM, and in the vacuole over a time course of 24 h after transformation. Cells showing PIN1-RFP both at PM and either at the ER or vacuole were ascertained as ER or vacuole positive, respectively. Bars = 10 μm.

Ten hours after transformation, we observed colocalization of PIN1-RFP with the inert ER marker HDEL:GFP in most of the transformed cells (87%) (Figure 3A and 3D), implying high levels of newly synthesized PIN proteins, whereas 16 h after transformation, in most analyzed cells, PIN1-RFP was absent from the ER and solely visible (at the given confocal setting) at the plasma membrane (Figure 3B and 3D) where it is active [21]. Afterwards, the percentage of cells with a strong PIN1-RFP signal at the plasma membrane diminished over time (Figure 3D). This decrease in PIN1 localization at the plasma membrane correlated with an increase of the PIN1-RFP occurrence in the vacuole (Figure 3C and 3D), hinting at a PIN1 turnover by lytic degradation [45].

**Figure 4:**
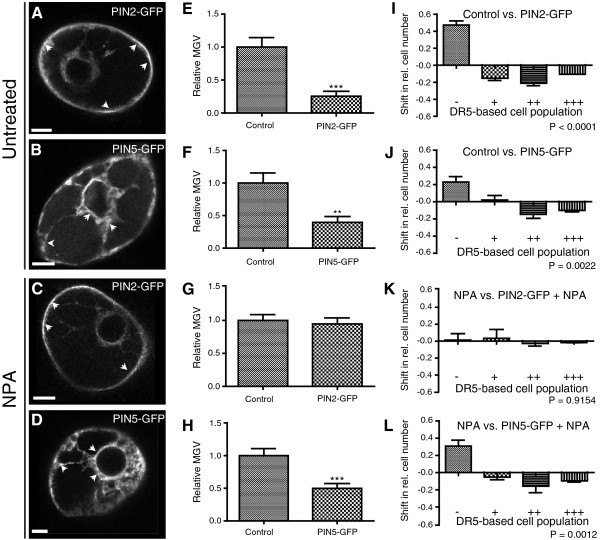
**Effect of PIN protein activity on cellular auxin homeostasis.** (**A**) Preferential PIN2-GFP localization at the plasma membrane. (**B**) Typical perinuclear ER localization of PIN5-GFP. (**C**,**D**) PIN2-GFP and PIN5-GFP localization is not affected in the presence of the auxin transport inhibitor NPA (application with 10 μM NPA-enriched medium). Arrowheads depict preferential PIN2 and PIN5 localizations at the PM and ER, respectively. Bars = 10 μm. (**E**,**I**,**F**,**J**) Cells co-transformed with *DR5rev*:*mRFP* and either *35S*:*PIN2*-*GFP* or *35S*:*PIN5*-*GFP* had a lower DR5rev:mRFP signal intensity than the control cells expressing *DR5rev*:*mRFP* and the inert ER marker *35S*:*HDEL*-*GFP*. (**G**,**H**,**K**,**L**) In the presence of NPA, PIN5-GFP but not PIN2-GFP expression decreases DR5rev:mRFP signal intensity. (**E**-**H**) Graphs represent the relative average mean gray values (MGV) of the DR5rev:mRFP signal intensity. Error bars represent standard error (n = 60). Statistical significance was evaluated with the unpaired student T-test (* P < 0.05, ** P < 0.01, *** P < 0.0001). (**I**-**L**) Graphs depict the change in relative number of transformed cells displaying a low (-), medium (+), high (++), and very high (+++) DR5rev:mRFP signal intensity between the two samples (for detailed description of the quantification, see Additional file 1: Figure S2). Error bars represent standard error (n = 3 repetitions with at least 50 counted cells). Statistical significance was evaluated with the ANOVA test; the P- value is indicated.

Altogether, PIN1 proteins displayed a pronounced localization and, presumably, high activity at the plasma membrane between 16 h-18 h after transformation. At that time point (17 h after transformation), also other PIN proteins, such as PIN2-GFP and PIN5-GFP, strongly localized at the plasma membrane and ER (Figure 4A and 4B), respectively. We conclude 16 h to 18 h as a suitable time frame for the analysis of PIN auxin carrier activity.

### Activity of the PIN auxin transport proteins affects auxin signaling

Next, we investigated the effect of the PIN auxin carrier activity on the cellular auxin signaling using the DR5- and single-cell-based system. Cells cotransformed with *DR5rev*:*mRFP* and *35S*:*PIN2*-*GFP* had a lower DR5rev:mRFP signal intensity than the control cells expressing *DR5rev*:*mRFP* and the inert ER marker *35S*:*HDEL*-*GFP* (Figure 4A, 4E and 4I). These observations are in agreement with the PIN induced decrease in cellular auxin accumulation (Figure 1A–I) and indicate a decreased auxin signaling due to the enhanced PIN2-auxin efflux carrier activity at the plasma membrane.

PIN5 is an ER localized auxin carrier described to facilitate auxin transport from the cytosol into the ER. This auxin sequestration into the ER presumably reduces the availability of auxin for nuclear auxin signaling [14]. In agreement with these assumptions, the *PIN5GFP* expression caused a significant decrease in the DR5rev:mRFP signal intensity (Figure 4B, 4F and 4J).

We conclude that the DR5- and single-cell-based system can be used to indirectly monitor the activity of plasma membrane and ER localized PIN auxin transporters.

### Auxin transport inhibitor NPA inhibits PIN2, but not PIN5 action in the single cell system

As described above, the expression of *PIN2GFP* facilitates the auxin efflux from cells and, hence, lowers the levels of intracellular auxin signaling (Figure 4E and 4I). After treatment with the auxin transport inhibitor NPA, *PIN2* expression did not decrease auxin signaling compared to NPA treated *HDELGFP* expressing control cells (Figure 4G and 4K). Importantly, NPA did not visibly affect the transient PIN2 localization at the plasma membrane (Figure 4C), indicating that our single-cell-based system monitors the inhibitory effect of NPA on the auxin transport activity of PIN2 [21,23].

In contrast to PIN2, the *PIN5GFP* expression (Figure 4D) reduced the auxin signaling even in the presence of NPA (Figure 4H and 4L). This difference in NPA sensitivity indicates that the mechanisms of NPA action and/or auxin transport mechanisms of PIN2 and PIN5 are distinct. This finding is in agreement with a NPA binding activity at the plasma membrane [46]. To further assess the NPA insensitivity of putative auxin carriers at the ER, we analyzed PILS putative auxin carrier activity in our system. PILS5 localizes to the ER and was recently described to decrease nuclear auxin signaling presumably due to auxin sequestration into the ER [17]. Similar to PIN5, we observed a PILS5 dependent decrease in DR5 signaling in the presence of NPA (Additional file 1: Figure S4). Hence, we assume that NPA inhibits intercellular, but not intracellular auxin transport at the ER in BY-2 cell cultures.

We conclude that the single-cell-based system is a sensitive approach not only to monitor the carrier-driven auxin homeostasis, but also to assess the auxin carrier sensitivity to auxin transport inhibitors. These data demonstrate that our transient approach can be used to investigate the genetic or pharmacological interferences with auxin carrier function.

### WAT1 protein activity affects cellular auxin homeostasis

**Figure 5:**
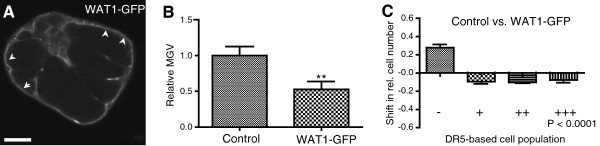
**Effect of WAT1:GFP protein activity on cellular auxin homeostasis.** (**A**) Preferential WAT1:GFP localization at the tonoplast (arrowheads). Bar = 10 μm. (b,c) Lower DR5rev:mRFP intensity in cells expressing *35S*:*WAT1*:*GFP* than control cells co-expressing *DR5rev*:*mRFP* and *35S*:*GFP*:*GFP*. (**B**) Graph represents the relative average mean gray values (MGV) of the DR5rev:mRFP signal intensity. Error bars represent standard error (n = 60). Statistical significance was evaluated with the unpaired student T-test (* P < 0.05, ** P < 0.01, *** P < 0.0001). (**C**) Graph depicts the change in relative number of transformed cells displaying a low (-), medium (+), high (++), and very high (+++) DR5rev:mRFP signal intensity between the two samples (for detailed description of the quantification, see Additional file 1: Figure S2). Error bars represent standard error (n = 3 repetitions with at least 50 counted cells). Statistical significance was evaluated with the ANOVA test; the P-value is indicated.

To assess whether the approach is suitable to monitor also the activity of other putative transporters, we co-expressed *DR5rev*:*mRFP* with *WALLS ARE THIN1* (*WAT1*) (Figure 5A). WAT1 is a tonoplast-localized transmembrane protein that belongs to the drug/metabolite transporter superfamily. WAT1 activity has an impact on auxin homeostasis by affecting tryptophan and/or auxin metabolism via an unknown mechanism [47]. We used our single-cell-based system to investigate whether WAT1 affects the cellular auxin signaling in BY-2 cells. Cells co-transformed with *DR5rev*:*mRFP* and *35S*:*WAT1*:*GFP* had a lower DR5rev:mRFP signal intensity than control cells expressing *DR5rev*:*mRFP* and *35S*:*GFP*:*GFP* (Figure 5B and 5C), implying a negative effect of WAT1 protein activity on nuclear auxin signaling. These data show that our single-cell-based system visualizes the effect of WAT1 on cellular auxin homeostasis and that it could be in principle used to indirectly asses the activity of a wide range of carrier proteins.

### Indirect visualization of the cellular cytokinin homeostasis

**Figure 6:**
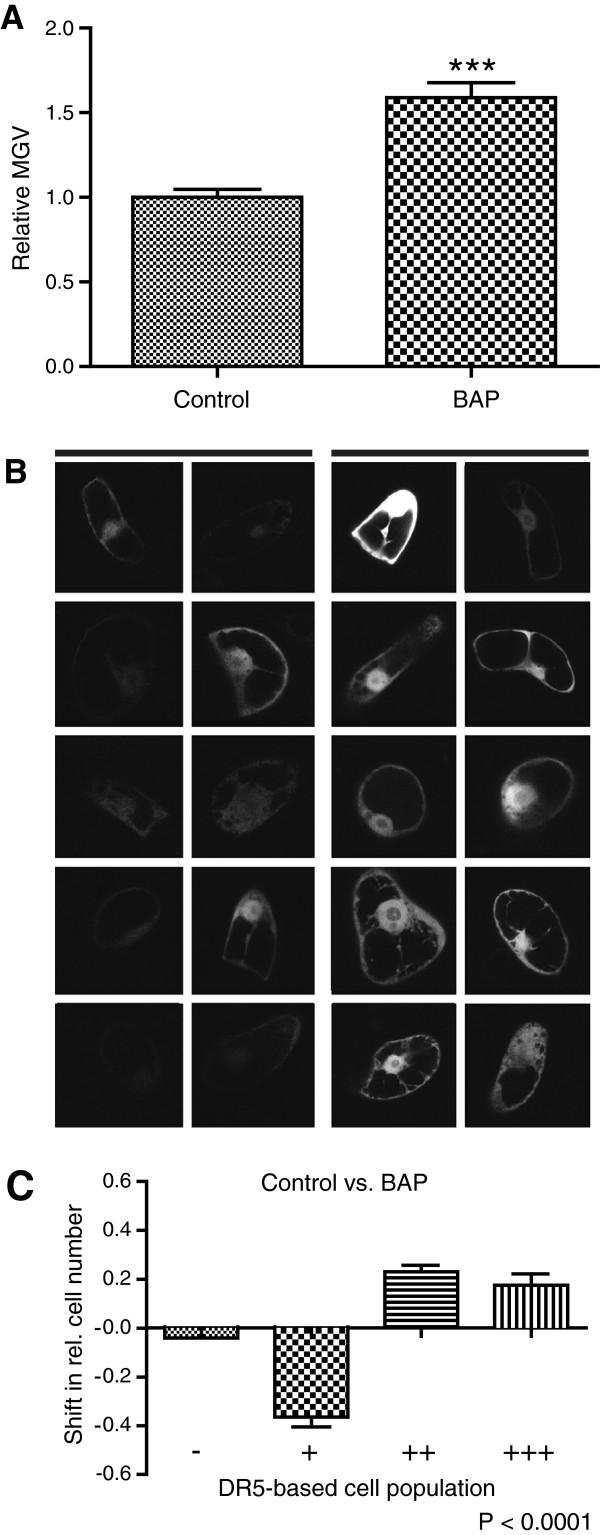
**Effect of exogenous 6-benzylaminopurine (BAP) on cellular cytokinin signaling.** BAP treatment (application with 10 μM BAP-enriched medium) led to an increased TCS:GFP signaling compared to transformants maintained in the standard cultivation medium (control). (**A**) Graph represents the relative average mean gray values (MGV) of the TCS:GFP transformed BY-cells. Error bars represent standard error (n = 60). Statistical significance was evaluated with the unpaired student T-test (* P < 0.05, ** P < 0.01, *** P < 0.0001). (**B**) Representative pictures show the TCS:GFP signal intensities of 10 untreated (left) and BAP treated (right) transformed cells. (**C**) Graph depicts the change in relative number of transformed cells displaying a low (-), medium (+), high (++), and very high (+++) TCS:GFP signal intensity between the two samples (for detailed description of the quantification, see Additional file 1: Figure S2). Error bars represent standard error (n = 3 repetitions with at least 50 counted cells). Statistical significance was evaluated with the ANOVA test; the *P*-value is indicated.

As the single-cell-based system enables the indirect monitoring of carrier driven cellular auxin homeostasis, we tested whether the method could be used analogously for other hormonal pathways. The synthetic cytokinin-responsive promoter TWO-COMPONENT-OUTPUT-SENSOR (TCS):GFP was expressed transiently in tobacco BY-2 cells for indirect visualization of the cellular cytokinin signaling [48]. The distribution characteristics of the cells transiently transformed with TCS:GFP were similar to those previously observed for DR5rev:mRFP. Analogously, we measured the average MGV of the transformed cell population and furthermore categorized the cells in subpopulations with very strong, strong, medium, and low TCS:GFP signal intensity (Figure 6A–C). The TCS:GFP activity was higher in transformants treated with 6-benzylaminopurine (BAP), a native aromatic cytokinin [1], than in those grown in standard cultivation medium (Figure 6A and 6B), suggesting an enhanced cytokinin signaling in BY-2 cells upon cytokinin application.

These results suggest that our single-cell-based system could eventually be extended to other applications, such as the indirect visualization of cellular cytokinin signaling.

## Discussion and conclusion

The phytohormone auxin plays a key role in many aspects of plant growth and development. The cellular auxin content is tightly controlled by local auxin metabolism (biosynthesis, conjugation/deconjugation, or oxidation) and auxin transport facilitators [5,28,49-52]. Whereas the complex interplay of these factors still needs to be unraveled, it is clear that various transporters have pronounced effects on the cellular auxin homeostasis [7,9,17,21,47,53]. Furthermore, the steady release of new annotated genomes increases the number of putative auxin carriers and enables the study of their molecular evolution. The scientific progress in auxin carrier identification emphasizes the growing demand for suitable approaches to assess carrier-driven cellular auxin homeostasis.

Here, we present a single-cell-based system that allows us to monitor qualitative differences in nuclear auxin signaling between two samples of interest. Thanks to this easy approach, carrier-driven auxin homeostasis and its sensitivity to auxin transport inhibitors can be visualized. The transient approach enables (possibly in combination with automated imaging systems) medium-to-high throughput work flows that can be used for chemical genomic or gain- and loss-of-function screens. The DR5rev:mRFP signal intensity can be easily estimated by measuring the mean grey values. Ratiometric imaging of DR5 signaling and a constitutive (auxin independent) marker could furthermore increase the sensitivity of the approach. Also the usage of the so-called novel auxin signaling sensor (Aux/IAA-based) termed DII-VENUS [54] could be useful to improve the temporal resolution of the system, because DII is not based on gene regulation, but on auxin-dependent protein degradation. For high-throughput work flows, automation, such as qRT-PCR or luciferase-based detection, might be most beneficial.

Various transient expression approaches, such as gold particle bombardment, micro-injection, polyethylene glycol (PEG)-mediated DNA uptake, and electroporation of protoplasts [55-58] have been successfully used to transiently transform plant cells. Whereas transient transformation of protoplasts has been proven to be highly efficient in high-throughput work flows, particle bombardment of plant cells might be preferable for investigating auxin carrier activity, because it does not affect the cell wall integrity that might be required for auxin carrier trafficking and function [59-61]. Accordingly, here we utilized particle bombardment of BY-2 cells as a transient transformation system to establish a single-cell-based system to monitor cellular auxin homeostasis.

As a proof of concept, we investigated prominent PIN auxin efflux carriers and visualized their action on the cellular auxin signaling. In stably transformed BY-2 cell lines, we illustrate that PIN-dependent reduction in cellular accumulation of exogenous auxin correlates with decreased (DR5-based) nuclear auxin signaling. Using the single-cell-based system, we reveal the differential sensitivities of PIN2, PIN5 and PILS5 to the auxin transport inhibitor NPA. Under our experimental condition, NPA blocks PIN2 action at the plasma membrane, but does not diminish PIN5 and PILS5 function at the ER, indicating that auxin transport mechanisms at the plasma membrane and at the ER could be partially distinct. These findings are in agreement with the assumption that NPA action on auxin carrier activity might be restricted to the plasma membrane [46]. As such, NPA could be applied to distinguish between intercellular and intracellular auxin transport. We assume that the differential sensitivity of PIN2 and PIN5 to NPA indicate the suitability of the system for chemical genetic approaches.

Besides the analysis on PIN2, PIN5 and PILS5, we also confirmed that WAT1 negatively affects cellular auxin signaling. WAT1 localizes to the tonoplast and has been suggested to regulate cellular auxin homeostasis [47], possibly by sequestering a yet to be identified auxinic compound into the vacuole. How WAT1 affects auxin homeostasis is still unclear and the mechanism awaits in-depth characterization. Nevertheless, WAT1 activity could be visualized indirectly with our system that might be helpful to further characterize its functionality.

In summary, we established an easy and useful tool to visualize carrier activities that affect cellular auxin signaling. This complementary method bridges the gap between highly elaborated direct auxin transport assays and indirect approaches such as root hair-based visualization of carrier-driven cellular auxin homeostasis [9,14,20-25]. Given the transient nature of our approach, it allows, for instance, the rapid and systematic pre-screening of several mutant versions of an auxin carrier of interest. Subsequently, interesting candidates could be analyzed in depth in other, more elaborated systems.

This single-cell-based system could be also used to analyze other molecular components involved in auxin homeostasis, such as regulators of the auxin signaling or metabolism. Moreover, it could be eventually extended to investigate other hormonal pathways by means of different reporter constructs, such as the cytokinin-responsive element TCS:GFP [48]. However, compared to the DR5 auxin reporter, further in depth characterization of TCS:GFP activity in BY-2 cells is needed to use the system analogously.

## Methods

### Plant material and growth conditions

*Nicotiana tabacum* L. cv. Bright Yellow-2 cell line [62] was cultivated at 25°C in darkness on an orbital incubator at 150 rpm in liquid medium (3% sucrose, 4.3 g L^-1^ Murashige and Skoog salts, 100 mg L^-1^ inositol, 1 mg L^-1^ thiamin, 0.2 mg L^-1^ 2,4-dichlorophenoxyacetic acid (2,4-D) and 200 mg L^-1^ KH_2_PO_4_, pH 5.8) and subcultured weekly (50x dilution). The used constructs for transient BY-2 cell transformation have been described previously: *DR5rev*:*mRFP*[37], *XVE*::*PIN1*:*GFP*[38], *GVGPIN7*[21], *35S*:*PIN2GFP*[63], *35S*:*PIN5GFP*[14], *35S*:*PILS5*_*D*[17], *35S*:*HDELGFP*[64], *35S*:*PIN1RFP*[59], *35S*:*VP16IAA17mImII*[38], *35S*:*WAT1*:*GFP*[47], *35S*:*GFP*:*GFP*[47], and *TCS*:*GFP*[48]. Expression of *PIN1GFP* in *XVE*:*PIN1GFP*/*DR5*:*mRFP1* genes was induced by the addition of β-estradiol (1 μM, 48 h) and *PIN7* in *GVG*:*PIN7*/*DR5*:*mRFP* by the addition of dexamethasone (3 μM, 48 h) at the beginning of the subculture interval. The solvent DMSO (estradiol) or H_2_O (dexamethasone) were also added to control samples. We used *Arabidopsis thaliana* of ecotype Columbia 0 (Col-0). Seedlings were grown vertically on half Murashige and Skoog medium. Plants were grown under long-day (16 h light/8 h dark) conditions at 20–22°C. The *Arabidopsis thaliana* DR5rev:GFP line was described previously [30]. Treatment with 1 μM brassinolide for 18h was performed on 7 day old seedlings in liquid growth medium.

### Stable transformation of BY-2 cells

The basic transformation protocol of An [65] was used. For the transformation, BY-2 lines carrying *PIN1*:*GFP* gene, under the estradiol-inducible transactivator XVE, (XVE-PIN1:GFP) [38] or *PIN7* gene under dexamethasone-inducible promoter (line GVG-PIN7) [21] were used. Three-day-old BY-2 cells were co-incubated with *Agrobacterium tumefaciens* strain GV2260 carrying *DRrev5*:*mRFP* construct. Resulting double transformed lines were maintained in culture media containing 100 μg/mL kanamycin, 100 μg/mL hygromycin and 100 μg/ml cefotaxim.

### Verification of transgene expression using Quantitative Reverse Transcription Polymerase Chain Reaction (qRT-PCR)

Tobacco total RNA was extracted from stably transformed BY-2 cells (induction of expression GVG-PIN7 by the dexamethasone (3 μM, 24 h)) using SpectrumTM Plant Total RNA Kit (Sigma - Aldrich) and treated with DNAse from DNA-freeTM Kit (Ambion). M-MLV Reverse Transcriptase (H-) (Promega) was used to generate cDNA, according to the manufacturer's instructions. qPCR was performed using DyNAmoTM Flash SYBR® Green qPCR Kit (Finnzymes). Specific primers: AtPIN7 forward 5^′^-GGGAAGAAGAGTCGGAGAG-3^′^, reverse 5^′^-AAGAGCCCAAATGAGACCAA-3^′^; Ta = 56°C. Resulting values are expressed as a ratio of relative expression of particular gene in induced cells against relative expression of this gene in non-induced cells. Actin was used as reference gene.

### Auxin accumulation measurements

Auxin accumulation in 2-day-old cells was measured using radioactively labelled auxins according to [66], as modified by [21]. Treatments were replicated at least three times and the average values (± standard errors) were expressed as pmols of the particular auxin accumulated per million cells. At the beginning of the accumulation assay ^3^H]NAA (20 Ci mmoL^-1^; American Radiolabeled Chemicals, Inc., St Louis, MO, USA) (as a good substrate of auxin efflux carriers) was added to the PIN1-GFP induced BY-2 cell line XVE-PIN1:GFP/DR5rev:mRFP (non-induced line was used as a control) to give a final concentration 2nM of ^3^H]NAA.

### Transient transformation of BY-2 cells

Adjusted from previously described procedures [39,40] 10 ml of three-day-old cells were harvested on filter paper by vacuum filtration and kept on plates with BY-2 medium solidified with 0,6% agar. The cells were transformed via particle bombardment with a PDS 1000/He biolistic system (Bio-Rad) according to the manufacturer's instructions (http://www.bio-rad.com/webroot/web/pdf/lsr/literature/Bulletin_9075.pdf). To coat the gold particles with DNA, 2 μl of plasmid DNA (if not indicated differently, 0.05 μg/μl of each construct to transform) was added to 6.25 μl of 1.6-μm diameter gold particles (dissolved in 50% glycerol). The suspension was supplemented with 2.5 μl spermidine (0.1 M stock solution) and 6.25 μl CaCl_2_ (2.5 M stock solution). For *35S*:*PIN2GFP*, *35S*:*PIN5GFP*, *35S*:*HDEL*:*GFP*, and *35S*:*VP16IAA17mImII*, 0.1 μg/μl was used for the transformation. The particles were pelleted by centrifugation, washed twice with 70% and 100% ethanol and, subsequently, resuspended in 10 μl of 100% ethanol. Cells were bombarded under a pressure of 1100 psi. Pharmacological treatments were done by applying 0.5 ml of BY-2 growth medium, enriched with 10 μM NPA, 10 μM 6-benzylaminopurine (BAP) (Duchefa) or 1 μM brassinolide (BR) (Fuji Chemical Industries) directly after transformation. The plates were sealed with parafilm and kept in the dark for 18 h at 25°C. For microscopic analysis, cells were gently transferred (with a spatula) from the filter to a microscopy slide (in water) and subsequently covered with a cover slip. Samples were analyzed via confocal microscopy.

### Microscopy

Live-cell confocal microscopy was done with a Zeiss 710 microscope. Fluorescence signals for GFP (excitation 488 nm, emission peak 509 nm) and mRFP1 (excitation 561 nm, emission peak 607 nm) were detected. Sequential scanning was used for double labeling to avoid crosstalk between channels. The *DR5rev*:*mRFP* expression was evaluated by defining the mean gray value (MGV) of each imaged cell (middle sections). For each experiment, confocal settings were defined based on the DR5rev:mRFP signal of the control cells and remained unchanged during the respective experiment. Transformants were identified based on the fluorescence of both proteins, imaged with a 40x objective, and subdivided into four clusters (very low, low, medium, and high) according to the relative MGV (See also Additional file 1: Figure S2). Every experiment was done in triplicate (independent transformations) and for each condition, a total number of at least 60 transformed cells were imaged. The means and standard errors were calculated and the statistical significance (independence between the two populations) was obtained by student t-test (for the analysis of the MGV) and ANOVA analysis (for the subdivision into clusters).

## Abbreviations

BY-2: Bright Yellow-2; PIN: Pin-formed; ER: Endoplasmic reticulum; NPA: 1-naphthylphthalamic acid; PILS: PIN-likes; WAT1: Walls are thin1; ARF: Auxin response factor; TIR/AFB: Transport inhibitor repsonse/auxin F-box protein; AUX/LAX: Auxin resistant1/like aux1; GH3: Gretchen Hagen 3; mRFP: Monomeric red fluorescent protein; NAA: 1-Naphthylacetic acid; MGV: Mean gray value; GFP: Green flurorescent protein; TCS: Two-component-output-sensor; BAP: 6-benzylaminopurine; PEG: Polyethylene glycol.

## Competing interests

We certify that there is no conflict of interest with any financial organization regarding the data and material discussed in the manuscript.

## Authors’ contributions

EB and JKV conceived the project, EB carried out most of the experiments, ML and MP performed the auxin accumulation assays and imaged the stably transformed BY-2 cell lines. AM supplied the DR5rev:mRFP construct. EB, ML, MP, EZ, JP, AM, JF and JKV discussed the results, EB and JKV wrote the manuscript. All authors have read and approved the final manuscript.

## Supplementary Material

Additional file 1 **Barbez et al Supplementary information. Table S1.** Cotransformation efficiencies. The cotransformation efficiency was measured for two constructs transformed at several concentration ratios. Transformants were identified based on the presence of plasmid 1 and the percentage of cells carrying both plasmids was calculated. **Figure S1:** Correlation between PIN1-GFP and DR5rev:mRFP signal intensity. (A) Graph depicts relative *PIN7* expression levels of dexamethasone induced *GVG*-*PIN7* and non-induced cells analysed by quantitative-RT-PCR (n = 3). (B) Estradiol induced BY-2 cells show individual variability of *PIN1*-*GFP* expression. Cellular intensity of PIN1-GFP reveals a negative correlation between PIN1-GFP and DR5rev:mRFP signal intensity. Strongly *PIN1*-*GFP* expressing cells show a strong decrease in DR5rev:mRFP signal intensity (green arrowheads) compared to cells with weaker *PIN1*-*GFP* expression (red arrowheads). (C) Scatterplot depicts single cell mean gray value (MGV) of the PIN1-GFP and the corresponding DR5rev:mRFP fluorescent intensity (n = 178). **Figure S2.** DR5rev:mRFP signal intensity quantification. DR5rev:mRFP signal intensity is visualized by gray scale representation and the mean gray value (MGV) of each transformed cell is measured using Image J. The relative MGV of each cell is calculated according to the average MGV of the control sample. Individual relative MGV are depicted in the pictures. The transformed cell population of each sample is subdivided in 4 classes according to the relative MGV. Cells were scored as low (-) with a relative MGV below 0.5 (= 2^-1^), medium (+) with a relative MGV between 0.5 (= 2^-1^) and 1 (= 2^0^), high (++) with a relative MGV between 1 (= 2^0^) and 2 (= 2^1^) and very high (+++) with a mean grey value higher then 2 (= 2^1^). This evaluation visualizes the variability of DR5rev:RFP signal intensity within the transformed cell population. In the used confocal settings, most of the visualized cells clustered in the categories medium and strong. **Figure S3.** Effect of brassinolide on cellular auxin homeostasis. (A) *DR5rev*:*GFP* expression in the root tip of brassinolide (1 μM; 18 hours) treated and untreated *Arabidopsis thaliana* seedlings. Graph represents the relative average mean gray values (MGV) of DR5rev:GFP intensity. Error bars represent standard error (n > 20). (B) Representative pictures display DR5rev:GFP signal intensity of untreated (left) and brassinolide treated (right) seedlings. Color-code (black to white) depicts (low to high) DR5*rev*:GFP signal intensity. (C) Graph represents the relative average MGV of the *DR5rev*:*mRFP* transformed BY-cells. Error bars represent standard error (n > 50). Application with 1 μM brassinolide-enriched medium did not lead to a significant change in the average relative MGV of DR5rev:mRFP. Statistical significance was evaluated with the unpaired student T-test (* P < 0.05, ** P < 0.01, *** P < 0.0001). (D) Representative pictures show the DR5rev:mRFP signal intensities of 10 transformed control (left) and brassinolide treated (right) cells. (E) Graph depicts the change in relative number of transformed cells displaying a low (-), medium (+), high (++), and very high (+++) DR5rev:mRFP signal intensity between the two samples. For detailed description of the quantification, see Additional file 1: Figure S2. Brassinolide treatment (application with 1μM brassinolide-enriched medium) leads to a significant change in relative number of cells displaying a low, medium, high, and very high DR5rev:mRFP signal intensity indicating that brassinolide affects the variability of relative MGV within the transformed cell population. Error bars represent standard error (n = 3 repetitions with at least 50 counted cells). Statistical significance was evaluated with the ANOVA test; The P value is indicated. **Figure S4.** PILS5 sensitivity to NPA. In the presence of NPA, PILS5_D expression decreases DR5rev:mRFP signal intensity. (A) Graphs represent the relative average mean gray values (MGV) of the DR5rev:mRFP signal intensity. Error bars represent standard error (n = 60). Statistical significance was evaluated with the unpaired student T-test (* P < 0.05, ** P < 0.01, *** P < 0.0001). (B) Graphs depict the change in relative number of transformed cells displaying a low (-), medium (+), high (++), and very high (+++) DR5rev:mRFP signal intensity between the two samples (for detailed description of the quantification, see Additional file 1: Figure S2). Error bars represent standard error (n = 3 repetitions with at least 60 counted cells). Statistical significance was evaluated with the ANOVA test; the P-value is indicated. (PDF 5102 kb) Click here for file

## References

[B1] Perrot-Rechenmann C Cellular responses to auxin: division versus expansion Cold Spring Harb Perspect Biol 2010 2 5 a001446 10.1101/cshperspect.a001446 20452959 PMC2857164

[B2] Gray WM Kepinski S Rouse D Leyser O Estelle M Auxin regulates SCF(TIR1)-dependent degradation of AUX/IAA proteins Nature 2001 414 6861 271 276 10.1038/35104500 11713520

[B3] Dharmasiri N Dharmasiri S Estelle M The F-box protein TIR1 is an auxin receptor Nature 2005 435 7041 441 445 10.1038/nature03543 15917797

[B4] Kepinski S Leyser O Plant development: auxin in loops Curr Biol 2005 15 6 R208 R210 10.1016/j.cub.2005.03.012 15797014

[B5] Ruiz Rosquete M Barbez E Kleine-Vehn J Cellular Auxin Homeostasis: Gatekeeping Is Housekeeping Mol Plant 2011 5 4 772 786 22199236 10.1093/mp/ssr109

[B6] Zazimalova E Murphy AS Yang H Hoyerova K Hosek P Auxin transporters–why so many? Cold Spring Harb Perspect Biol 2010 2 3 a001552 10.1101/cshperspect.a001552 20300209 PMC2829953

[B7] Bennett MJ Marchant A Green HG May ST Ward SP Millner PA Walker AR Schulz B Feldmann KA Arabidopsis AUX1 gene: a permease-like regulator of root gravitropism Science 1996 273 5277 948 950 10.1126/science.273.5277.948 8688077

[B8] Luschnig C Gaxiola RA Grisafi P Fink GR EIR1, a root-specific protein involved in auxin transport, is required for gravitropism in Arabidopsis thaliana Genes Dev 1998 12 14 2175 2187 10.1101/gad.12.14.2175 9679062 PMC317016

[B9] Geisler M Blakeslee JJ Bouchard R Lee OR Vincenzetti V Bandyopadhyay A Titapiwatanakun B Peer WA Bailly A Richards EL Cellular efflux of auxin catalyzed by the Arabidopsis MDR/PGP transporter AtPGP1 Plant J 2005 44 2 179 194 10.1111/j.1365-313X.2005.02519.x 16212599

[B10] Tanaka H Dhonukshe P Brewer PB Friml J Spatiotemporal asymmetric auxin distribution: a means to coordinate plant development Cell Mol Life Sci 2006 63 24 2738 2754 17013565 10.1007/s00018-006-6116-5 PMC11136431

[B11] Katekar GF Geissler AE Auxin Transport Inhibitors: IV Evidence of a common mode of action for a proposed class of Auxin Transport Inhibitors: the phytotropins Plant Physiol 1980 66 6 1190 1195 10.1104/pp.66.6.1190 16661601 PMC440814

[B12] Rubery PH Carrier-mediated Auxin Transport Planta 1974 118 101 121 10.1007/BF00388387 24442257

[B13] Fujita T Sakaguchi H Hiwatashi Y Wagstaff SJ Ito M Deguchi H Sato T Hasebe M Convergent evolution of shoots in land plants: lack of auxin polar transport in moss shoots Evol Dev 2008 10 2 176 186 10.1111/j.1525-142X.2008.00225.x 18315811

[B14] Mravec J Skupa P Bailly A Hoyerova K Krecek P Bielach A Petrasek J Zhang J Gaykova V Stierhof YD Subcellular homeostasis of phytohormone auxin is mediated by the ER-localized PIN5 transporter Nature 2009 459 7250 1136 1140 10.1038/nature08066 19506555

[B15] Bosco CD Dovzhenko A Liu X Woerner N Rensch T Eismann M Eimer S Hegermann J Paponov IA Ruperti B The endoplasmic reticulum localized PIN8 is a pollen-specific auxin carrier involved in intracellular auxin homeostasis Plant J 2012 71 5 860 870 10.1111/j.1365-313X.2012.05037.x 22540348

[B16] Ding Z Wang B Moreno I Duplakova N Simon S Carraro N Reemmer J Pencik A Chen X Tejos R ER-localized auxin transporter PIN8 regulates auxin homeostasis and male gametophyte development in Arabidopsis Nat Commun 2012 3 941 22760640 10.1038/ncomms1941

[B17] Barbez E Kubes M Rolcik J Beziat C Pencik A Wang B Rosquete MR Zhu J Dobrev PI Lee Y A novel putative auxin carrier family regulates intracellular auxin homeostasis in plants Nature 2012 485 7396 119 122 10.1038/nature11001 22504182

[B18] Feraru E Vosolsobe S Feraru MI Petrášek J Kleine-Vehn J Evolution and structural diversification of PILS putative auxin carriers in plants Front Plant Traffic Transp 2012 10.3389/fpls.2012.00227 PMC3470039 23091477

[B19] Barbez E Kleine-Vehn J Divide Et Impera-cellular auxin compartmentalization Curr Opin Plant Biol 2012 10.1016/j.pbi.2012.10.005 23200033

[B20] Imhoff V Muller P Guern J Delbarre A Inhibitors of the carrier-mediated influx of auxin in suspension-cultured tobacco cells Planta 2000 210 4 580 588 10.1007/s004250050047 10787051

[B21] Petrasek J Mravec J Bouchard R Blakeslee JJ Abas M Seifertova D Wisniewska J Tadele Z Kubes M Covanova M PIN proteins perform a rate-limiting function in cellular auxin efflux Science 2006 312 5775 914 918 10.1126/science.1123542 16601150

[B22] Yang Y Hammes UZ Taylor CG Schachtman DP Nielsen E High-affinity auxin transport by the AUX1 influx carrier protein Curr Biol 2006 16 11 1123 1127 10.1016/j.cub.2006.04.029 16677815

[B23] Yang H Murphy AS Functional expression and characterization of Arabidopsis ABCB, AUX 1 and PIN auxin transporters in Schizosaccharomyces pombe Plant J 2009 59 1 179 191 10.1111/j.1365-313X.2009.03856.x 19309458

[B24] Lee SH Cho HT PINOID positively regulates auxin efflux in Arabidopsis root hair cells and tobacco cells Plant Cell 2006 18 7 1604 1616 10.1105/tpc.105.035972 16731587 PMC1488908

[B25] Ganguly A Lee SH Cho M Lee OR Yoo H Cho HT Differential auxin-transporting activities of PIN-FORMED proteins in Arabidopsis root hair cells Plant Physiol 2010 153 3 1046 1061 10.1104/pp.110.156505 20439545 PMC2899906

[B26] Jones AR Kramer EM Knox K Swarup R Bennett MJ Lazarus CM Leyser HM Grierson CS Auxin transport through non-hair cells sustains root-hair development Nat Cell Biol 2009 11 1 78 84 10.1038/ncb1815 19079245 PMC2635559

[B27] Ulmasov T Murfett J Hagen G Guilfoyle TJ Aux/IAA proteins repress expression of reporter genes containing natural and highly active synthetic auxin response elements Plant Cell 1997 9 11 1963 1971 9401121 10.1105/tpc.9.11.1963 PMC157050

[B28] Casimiro I Marchant A Bhalerao RP Beeckman T Dhooge S Swarup R Graham N Inze D Sandberg G Casero PJ Auxin transport promotes Arabidopsis lateral root initiation Plant Cell 2001 13 4 843 852 11283340 10.1105/tpc.13.4.843 PMC135543

[B29] Benkova E Michniewicz M Sauer M Teichmann T Seifertova D Jurgens G Friml J Local, efflux-dependent auxin gradients as a common module for plant organ formation Cell 2003 115 5 591 602 10.1016/S0092-8674(03)00924-3 14651850

[B30] Friml J Vieten A Sauer M Weijers D Schwarz H Hamann T Offringa R Jurgens G Efflux-dependent auxin gradients establish the apical-basal axis of Arabidopsis Nature 2003 426 6963 147 153 10.1038/nature02085 14614497

[B31] Dubrovsky JG Sauer M Napsucialy-Mendivil S Ivanchenko MG Friml J Shishkova S Celenza J Benkova E Auxin acts as a local morphogenetic trigger to specify lateral root founder cells Proc Natl Acad Sci U S A 2008 105 25 8790 8794 10.1073/pnas.0712307105 18559858 PMC2438385

[B32] Petersson SV Johansson AI Kowalczyk M Makoveychuk A Wang JY Moritz T Grebe M Benfey PN Sandberg G Ljung K An auxin gradient and maximum in the Arabidopsis root apex shown by high-resolution cell-specific analysis of IAA distribution and synthesis Plant Cell 2009 21 6 1659 1668 10.1105/tpc.109.066480 19491238 PMC2714926

[B33] Sorefan K Girin T Liljegren SJ Ljung K Robles P Galvan-Ampudia CS Offringa R Friml J Yanofsky MF Ostergaard L A regulated auxin minimum is required for seed dispersal in Arabidopsis Nature 2009 459 7246 583 586 10.1038/nature07875 19478783

[B34] Nakamura A Higuchi K Goda H Fujiwara MT Sawa S Koshiba T Shimada Y Yoshida S Brassinolide induces IAA5, IAA19, and DR5, a synthetic auxin response element in Arabidopsis, implying a cross talk point of brassinosteroid and auxin signaling Plant Physiol 2003 133 4 1843 1853 10.1104/pp.103.030031 14605219 PMC300737

[B35] Nemhauser JL Feldman LJ Zambryski PC Auxin and ETTIN in Arabidopsis gynoecium morphogenesis Development 2000 127 18 3877 3888 10952886 10.1242/dev.127.18.3877

[B36] Gallavotti A Long JA Stanfield S Yang X Jackson D Vollbrecht E Schmidt RJ The control of axillary meristem fate in the maize ramosa pathway Development 2010 137 17 2849 2856 10.1242/dev.051748 20699296 PMC2938917

[B37] Marin E Jouannet V Herz A Lokerse AS Weijers D Vaucheret H Nussaume L Crespi MD Maizel A miR390, Arabidopsis TAS3 tasiRNAs, and their AUXIN RESPONSE FACTOR targets define an autoregulatory network quantitatively regulating lateral root growth Plant Cell 2010 22 4 1104 1117 10.1105/tpc.109.072553 20363771 PMC2879756

[B38] Lankova M Smith RS Pesek B Kubes M Zazimalova E Petrasek J Hoyerova K Auxin influx inhibitors 1-NOA, 2-NOA, and CHPAA interfere with membrane dynamics in tobacco cells J Exp Bot 2010 61 13 3589 3598 10.1093/jxb/erq172 20595238 PMC2921198

[B39] Sanford JC Klein TM Wolf ED Allen N Delivery of substances into cells and tissues using a particle bombardement process UPST 1987 5 1 27 37 10.1080/02726358708904533

[B40] Klein TM Harper EC Svab Z Sanford JC Fromme ME Maliga P Stable genetic transformation of intact Nicotiana cells by the particle bombardement process Proc Natl Acad Sci USA 1988 85 22 8502 8505 10.1073/pnas.85.22.8502 16593993 PMC282486

[B41] Tiwari SB Hagen G Guilfoyle T The roles of auxin response factor domains in auxin-responsive transcription Plant Cell 2003 15 2 533 543 10.1105/tpc.008417 12566590 PMC141219

[B42] Li Y Hagen G Guilfoyle TJ An Auxin-Responsive Promoter Is Differentially Induced by Auxin Gradients during Tropisms Plant Cell 1991 3 11 1167 1175 12324587 10.1105/tpc.3.11.1167 PMC160083

[B43] Murphy AS Hoogner KR Peer WA Taiz L Identification, purification, and molecular cloning of N-1-naphthylphthalmic acid-binding plasma membrane-associated aminopeptidases from Arabidopsis Plant Physiol 2002 128 3 935 950 10.1104/pp.010519 11891249 PMC152206

[B44] Boutte Y Crosnier MT Carraro N Traas J Satiat-Jeunemaitre B The plasma membrane recycling pathway and cell polarity in plants: studies on PIN proteins J Cell Sci 2006 119 Pt 7 1255 1265 16522683 10.1242/jcs.02847

[B45] Kleine-Vehn J Leitner J Zwiewka M Sauer M Abas L Luschnig C Friml J Differential degradation of PIN2 auxin efflux carrier by retromer-dependent vacuolar targeting Proc Natl Acad Sci U S A 2008 105 46 17812 17817 10.1073/pnas.0808073105 19004783 PMC2584678

[B46] Cox DN Muday GK NPA binding activity is peripheral to the plasma membrane and is associated with the cytoskeleton Plant Cell 1994 6 12 1941 1953 11536654 10.1105/tpc.6.12.1941 PMC160574

[B47] Ranocha P Denance N Vanholme R Freydier A Martinez Y Hoffmann L Kohler L Pouzet C Renou JP Sundberg B Walls are thin 1 (WAT1), an Arabidopsis homolog of Medicago truncatula NODULIN21, is a tonoplast-localized protein required for secondary wall formation in fibers Plant J 2010 63 3 469 483 10.1111/j.1365-313X.2010.04256.x 20497379

[B48] Muller B Sheen J Cytokinin and auxin interaction in root stem-cell specification during early embryogenesis Nature 2008 453 7198 1094 1097 10.1038/nature06943 18463635 PMC2601652

[B49] Blakeslee JJ Peer WA Murphy AS Auxin transport Curr Opin Plant Biol 2005 8 5 494 500 10.1016/j.pbi.2005.07.014 16054428

[B50] Ljung K Hull AK Celenza J Yamada M Estelle M Normanly J Sandberg G Sites and regulation of auxin biosynthesis in Arabidopsis roots Plant Cell 2005 17 4 1090 1104 10.1105/tpc.104.029272 15772288 PMC1087988

[B51] Woodward AW Bartel B Auxin: regulation, action, and interaction Ann Bot (Lond) 2005 95 5 707 735 10.1093/aob/mci083 PMC4246732 15749753

[B52] Grunewald W Friml J The march of the PINs: developmental plasticity by dynamic polar targeting in plant cells EMBO J 2010 29 16 2700 2714 10.1038/emboj.2010.181 20717140 PMC2924653

[B53] Krouk G Lacombe B Bielach A Perrine-Walker F Malinska K Mounier E Hoyerova K Tillard P Leon S Ljung K Nitrate-regulated auxin transport by NRT1.1 defines a mechanism for nutrient sensing in plants Dev Cell 2010 18 6 927 937 10.1016/j.devcel.2010.05.008 20627075

[B54] Brunoud G Wells DM Oliva M Larrieu A Mirabet V Burrow AH Beeckman T Kepinski S Traas J Bennett MJ A novel sensor to map auxin response and distribution at high spatio-temporal resolution Nature 2012 482 7383 103 106 10.1038/nature10791 22246322

[B55] Schnorf M Neuhaus-Url G Galli A Iida S Potrykus I Neuhaus G An improved approach for transformation of plant cells by microinjection: molecular and genetic analysis Transgenic Res 1991 1 1 23 30 10.1007/BF02512993 1668908

[B56] Takeuchi Y Dotson M Keen NT Plant transformation: a simple particle bombardment device based on flowing helium Plant Mol Biol 1992 18 4 835 839 10.1007/BF00020031 1558958

[B57] Datta K Datta SK Transformation of rice via PEG-mediated DNA uptake into protoplasts Methods Mol Biol 1999 111 335 347 10080999 10.1385/1-59259-583-9:335

[B58] Niemes S Langhans M Viotti C Scheuring D San Wan Yan M Jiang L Hillmer S Robinson DG Pimpl P Retromer recycles vacuolar sorting receptors from the trans-Golgi network Plant J 2010 61 107 121 10.1111/j.1365-313X.2009.04034.x 19796370

[B59] Robert S Kleine-Vehn J Barbez E Sauer M Paciorek T Baster P Vanneste S Zhang J Simon S Covanova M ABP1 mediates auxin inhibition of clathrin-dependent endocytosis in Arabidopsis Cell 2010 143 1 111 121 10.1016/j.cell.2010.09.027 20887896 PMC3503507

[B60] Wabnik K Kleine-Vehn J Balla J Sauer M Naramoto S Reinohl V Merks RM Govaerts W Friml J Emergence of tissue polarization from synergy of intracellular and extracellular auxin signaling Mol Syst Biol 2010 6 447 21179019 10.1038/msb.2010.103 PMC3018162

[B61] Feraru E Feraru MI Kleine-Vehn J Martiniere A Mouille G Vanneste S Vernhettes S Runions J Friml J PIN polarity maintenance by the cell wall in Arabidopsis Curr Biol 2011 21 4 338 343 10.1016/j.cub.2011.01.036 21315597

[B62] Nagata T Nemoto Y Hasezawa S Tobacco BY-2 cell line as the "HeLa" cells in the cell biology of higher plants Int Rev Cytol 1992 132 1 30

[B63] Abas L Benjamins R Malenica N Paciorek T Wisniewska J Moulinier-Anzola JC Sieberer T Friml J Luschnig C Intracellular trafficking and proteolysis of the Arabidopsis auxin-efflux facilitator PIN2 are involved in root gravitropism Nat Cell Biol 2006 8 3 249 256 10.1038/ncb1369 16489343

[B64] Langhans M Marcote MJ Pimpl P Virgili-Lopez G Robinson DG Aniento F In vivo trafficking and localization of p24 proteins in plant cells Traffic 2008 9 5 770 785 10.1111/j.1600-0854.2008.00719.x 18266912

[B65] An G High efficiency transformation of cultured tobacco cells Plant Physiol 1985 79 568 570 10.1104/pp.79.2.568 16664453 PMC1074928

[B66] Delbarre AMP Imhoff V Guern J Comparison of mechanisms controlling uptake and accumulation of 2,4-dichlorophenoxy acetic acid, naphthalene-1-acetic acid, and indole-3-acetic acid in suspension-cultured tobacco cells Planta 1996 192 538 541 10.1007/BF00262639 28321663

