# Supplementary material for: Single-cell-based system to monitor carrier driven cellular auxin homeostasis
Source: BMC Plant Biol. 2013 Feb 4;13:20. doi: 10.1186/1471-2229-13-20 (PMC3598821; doi:10.1186/1471-2229-13-20)
Supplement: Additional file 1 — Barbez et al Supplementary information. Table S1. Cotransformation efficiencies. The cotransformation efficiency was measured for two constructs transformed at several concentration ratios. Transformants were identified based on the presence of plasmid 1 and the percentage of cells carrying both plasmids was calculated. Figure S1: Correlation between PIN1-GFP and DR5rev:mRFP signal intensity. (A) Graph depicts relative PIN7 expression levels of dexamethasone induced GVG-PIN7 and non-induced cells analysed by quantitative-RT-PCR (n = 3). (B) Estradiol induced BY-2 cells show individual variability of PIN1-GFP expression. Cellular intensity of PIN1-GFP reveals a negative correlation between PIN1-GFP and DR5rev:mRFP signal intensity. Strongly PIN1-GFP expressing cells show a strong decrease in DR5rev:mRFP signal intensity (green arrowheads) compared to cells with weaker PIN1-GFP expression (red arrowheads). (C) Scatterplot depicts single cell mean gray value (MGV) of the PIN1-GFP and the corresponding DR5rev:mRFP fluorescent intensity (n = 178). Figure S2. DR5rev:mRFP signal intensity quantification. DR5rev:mRFP signal intensity is visualized by gray scale representation and the mean gray value (MGV) of each transformed cell is measured using Image J. The relative MGV of each cell is calculated according to the average MGV of the control sample. Individual relative MGV are depicted in the pictures. The transformed cell population of each sample is subdivided in 4 classes according to the relative MGV. Cells were scored as low (-) with a relative MGV below 0.5 (= 2-1), medium (+) with a relative MGV between 0.5 (= 2-1) and 1 (= 20), high (++) with a relative MGV between 1 (= 20) and 2 (= 21) and very high (+++) with a mean grey value higher then 2 (= 21). This evaluation visualizes the variability of DR5rev:RFP signal intensity within the transformed cell population. In the used confocal settings, most of the visualized cells clustered in the categories medium and stron [file 1471-2229-13-20-S1.pdf]

Supplemental Table S1

**concentration plasmid 1 (µg/µl)**	**concentration plasmid 2 (µg/µl)**	**cotransformation efficiency**
0.05	0.01	32/40 (80%)
0.05	0.05	39/43 (91%)
0.05	0.1	40/40 (100%)

Table S1. Cotransformation efficiencies.

The cotransformation efficiency was measured for two constructs transformed at several concentration ratios. Transformants were identified based on the presence of plasmid 1 and the percentage of cells carrying both plasmids was calculated.

Supplemental Figure S1

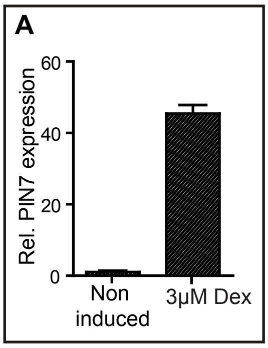

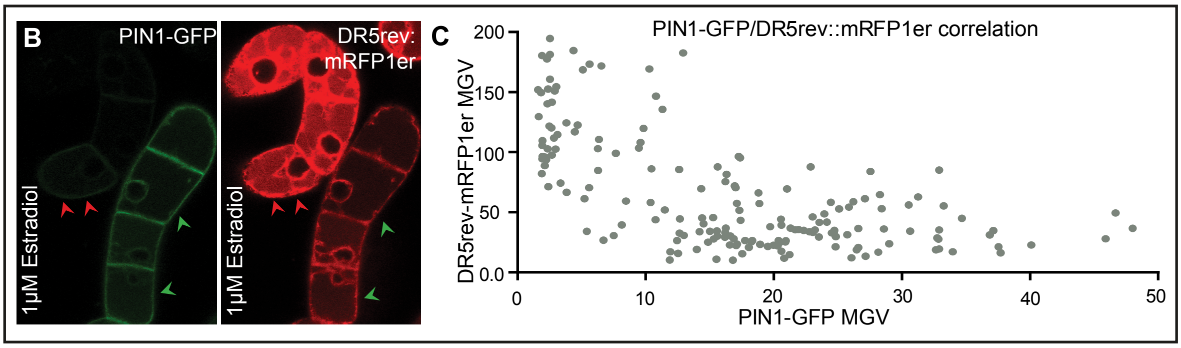

Figure S1: Correlation between PIN1-GFP and DR5rev:mRFP signal intensity.

(A) Graph depicts relative *PIN7* expression levels of dexamethasone induced *GVG-PIN7* and non-induced cells analysed by quantitative-RT-PCR (n=3). **(B)** Estradiol induced BY-2 cells shows individual variability of *PIN1-GFP* expression. Cellular intensity of PIN1-GFP reveals a negative correlation between PIN1-GFP and DR5rev:mRFP signal intensity. Strongly *PIN1-GFP* expressing cells show a strong decrease *in* DR5rev:mRFP signal intensity (green arrow heads) compared to cells with weaker *PIN1-GFP* expression (red arrow heads). **(C)** Scatterplot depicts single cell mean gray value (MGV) of the PIN1-GFP and the corresponding DR5rev:mRFP fluorescent intensity (n=178).

Supplemental Figure S2

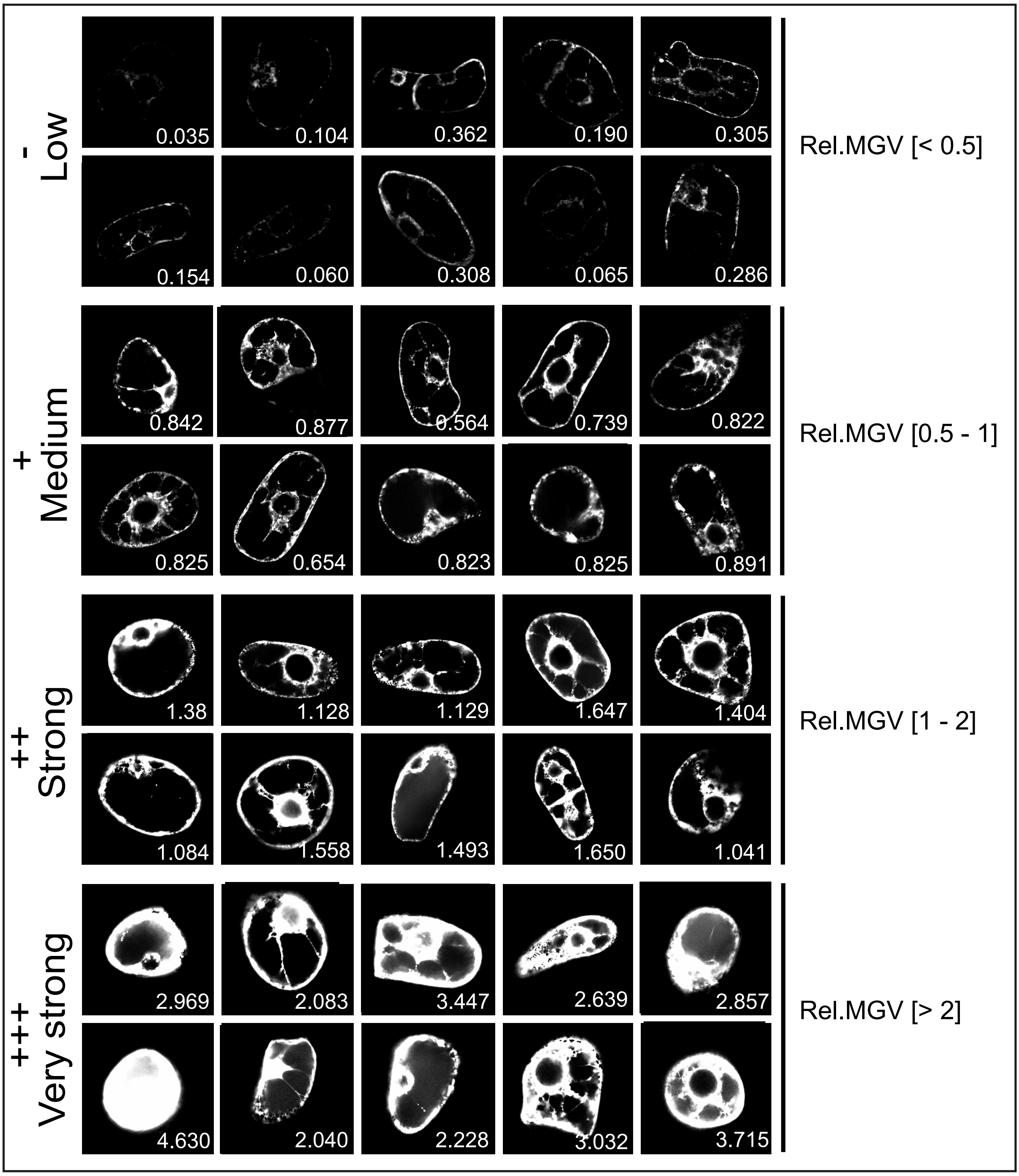

Figure S2. DR5rev:mRFP signal intensity quantification.

DR5rev:mRFP signal intensity is visualized by gray scale representation and the mean gray value (MGV) of each transformed cell is measured using Image J. The relative MGV of each cell is calculated according to the average MGV of the control sample. Individual relative MGV are depicted in the pictures.

The transformed cell population of each sample is subdivided in 4 classes according to the relative MGV. Cells were scored as low (-) with a relative MGV below 0.5 (= 2^{-1}), medium (+) with a relative MGV between 0.5 (= 2^{-1}) and 1 (= 2^{0}), high (++) with a relative MGV between 1 (= 2^{0}) and 2 (= 2^{1}) and very high (+++) with a mean grey value higher then 2 (= 2^{1}). This evaluation visualizes the variability of DR5rev:RFP1er signal intensity within the transformed cell population. In the used confocal settings, most of the visualized cells clustered in the categories medium and strong.

Supplemental Figure S3

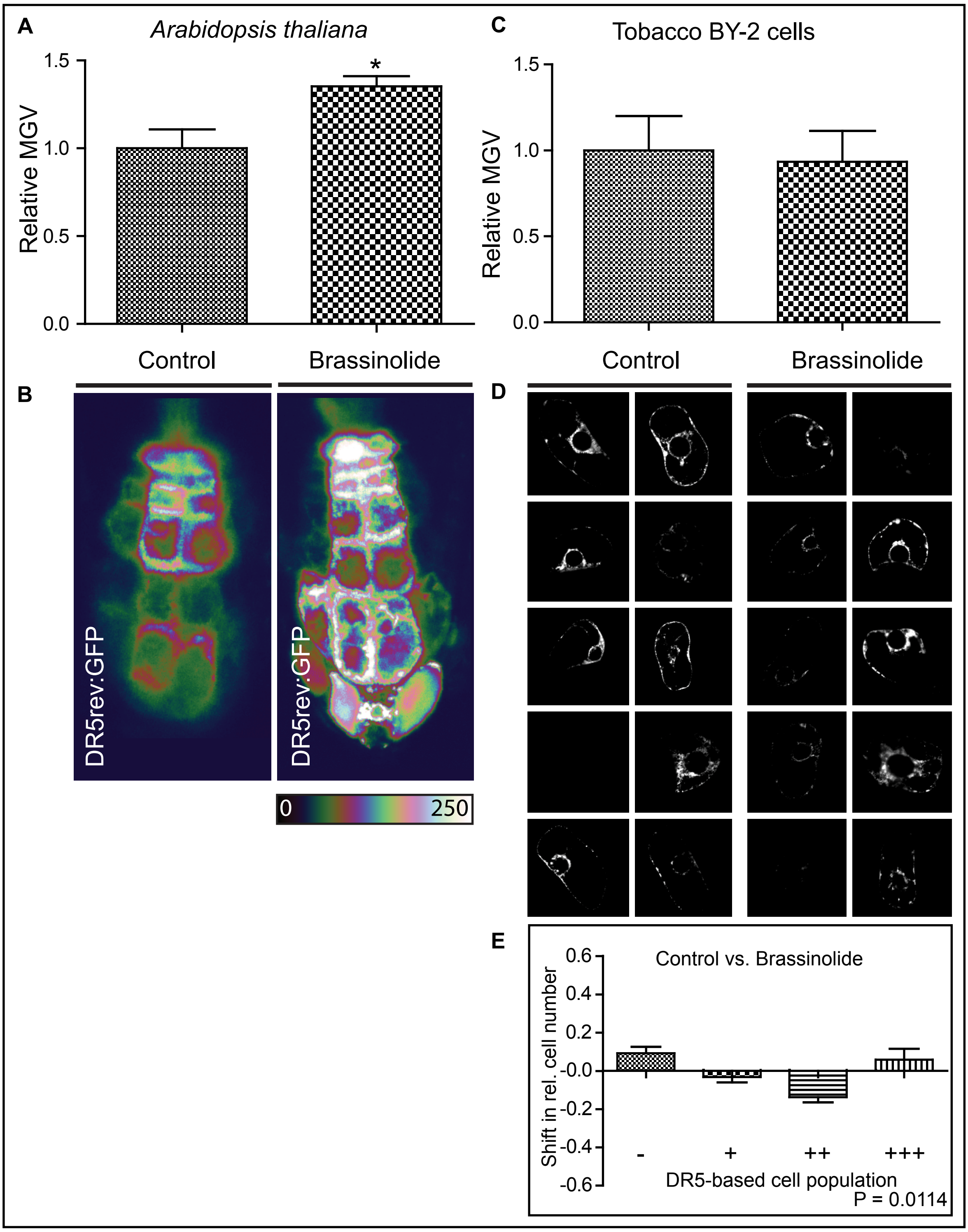

Figure S3. Effect of Brassinolide on cellular auxin homeostasis.

(A) *DR5rev:GFP* expression in the root tip of brassinolide (1µM; 18 hours) treated and untreated *Arabidopsis thaliana* seedlings. Graph represents the relative average mean gray values (MGV) of DR5rev:GFP intensity. Error bars represent standard error (n > 20). **(B)** Representative pictures display DR5rev:GFP signal intensity of untreated (left) and brassinolide treated (right) seedlings. Color-code (black to white) depicts (low to high) DR5*rev*:GFP signal intensity. **(C)** Graph represents the relative average MGV of the *DR5rev:mRFP* transformed BY-cells. Error bars represent standard error (n > 50). Application with 1µM brassinolide-enriched medium did not lead to a significant change in the average relative MGV of DR5rev:mRFP. Statistical significance was evaluated with the unpaired student T-test (* P < 0.05, ** P< 0.01, *** P < 0.0001). **(D)** Representative pictures show the DR5rev:mRFP signal intensities of 10 transformed control (left) and brassinolide treated (right) cells. **(E)** Graph depicts the change in relative number of transformed cells displaying a low (-), medium (+), high (++), and very high (+++) DR5rev:mRFP signal intensity between the two samples. For detailed description of the quantification, see Supplemental Figure S2. Brassinolide treatment (application with 1µM brassinolide-enriched medium) leads to a significant change in relative number of cells displaying a low, medium, high, and very high DR5rev:mRFP signal intensity indicating that brassinolide affects the variability of relative MGV within the transformed cell population. Error bars represent standard error (n=3 repetitions with at least 50 counted cells). Statistical significance was evaluated with the ANOVA test; The P value is indicated.

Supplemental Figure S4

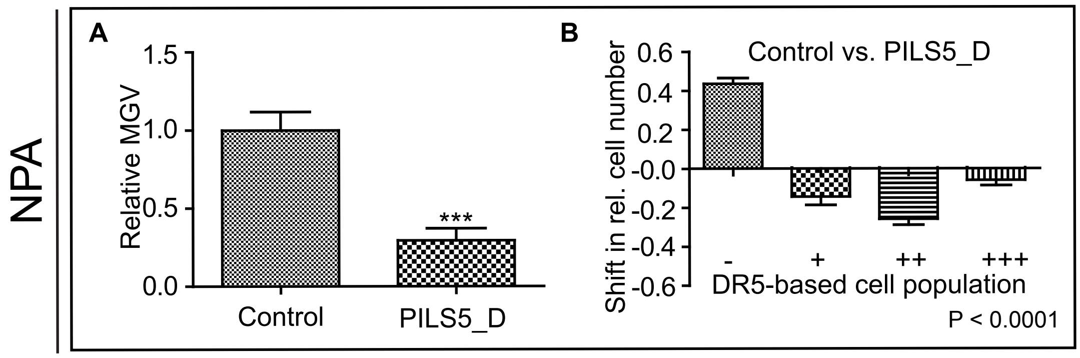

Figure S4. PILS5 sensitivity to NPA.

In the presence of NPA, PILS5_D expression decreases DR5rev:mRFP signal intensity. **(A)** Graphs represent the relative average mean gray values (MGV) of the DR5rev:mRFP signal intensity. Error bars represent standard error (n = 60). Statistical significance was evaluated with the unpaired student T-test (* $P < 0.05$, ** $P< 0.01$, *** $P < 0.0001$). **(B)** Graphs depict the change in relative number of transformed cells displaying a low (-), medium (+), high (++), and very high (+++) DR5rev:mRFP signal intensity between the two samples (for detailed description of the quantification, see Supplemental Figure S2). Error bars represent standard error (n=3 repetitions with at least 60 counted cells). Statistical significance was evaluated with the ANOVA test; the P- value is indicated.
